# A methodological study of genome-wide DNA methylation analyses using matched archival formalin-fixed paraffin embedded and fresh frozen breast tumors

**DOI:** 10.18632/oncotarget.14739

**Published:** 2017-01-19

**Authors:** Allyson C. Espinal, Dan Wang, Li Yan, Song Liu, Li Tang, Qiang Hu, Carl D. Morrison, Christine B. Ambrosone, Michael J. Higgins, Lara E. Sucheston-Campbell

**Affiliations:** ^1^ Department of Molecular and Cell Biology, Roswell Park Cancer Institute, Buffalo NY, USA; ^2^ Department of Biostatistics, Roswell Park Cancer Institute, Buffalo, NY, USA; ^3^ Department of Cancer Prevention and Control, Roswell Park Cancer Institute, Buffalo, NY, USA; ^4^ Department of Pathology, Roswell Park Cancer Institute, Buffalo, NY, USA; ^5^ College of Pharmacy, The Ohio State University, Columbus, OH, USA; ^6^ Department of Veterinary Biosciences, College of Veterinary Medicine, The Ohio State University, Columbus, OH, USA

**Keywords:** DNA methylation/epigenetics, breast cancer, estrogen receptor negative

## Abstract

**Background:**

DNA from archival formalin-fixed and paraffin embedded (FFPE) tissue is an invaluable resource for genome-wide methylation studies although concerns about poor quality may limit its use. In this study, we compared DNA methylation profiles of breast tumors using DNA from fresh-frozen (FF) tissues and three types of matched FFPE samples.

**Results:**

For 9/10 patients, correlation and unsupervised clustering analysis revealed that the FF and FFPE samples were consistently correlated with each other and clustered into distinct subgroups. Greater than 84% of the top 100 loci previously shown to differentiate ER+ and ER– tumors in FF tissues were also FFPE DML. Weighted Correlation Gene Network Analyses (WCGNA) grouped the DML loci into 16 modules in FF tissue, with ~85% of the module membership preserved across tissue types.

**Materials and Methods:**

Restored FFPE and matched FF samples were profiled using the Illumina Infinium HumanMethylation450K platform. Methylation levels (β-values) across all loci and the top 100 loci previously shown to differentiate tumors by estrogen receptor status (ER+ or ER−) in a larger FF study, were compared between matched FF and FFPE samples using Pearson's correlation, hierarchical clustering and WCGNA. Positive predictive values and sensitivity levels for detecting differentially methylated loci (DML) in FF samples were calculated in an independent FFPE cohort.

**Conclusions:**

FFPE breast tumors samples show lower overall detection of DMLs versus FF, however FFPE and FF DMLs compare favorably. These results support the emerging consensus that the 450K platform can be employed to investigate epigenetics in large sets of archival FFPE tissues.

## INTRODUCTION

DNA methylation, a heritable and modifiable epigenetic modification essential for development, is commonly disrupted in malignancies [1–3]. Due to its functional relevance, and the potential ability to modify DNA methylation, there is growing research focus on its relationship to cancer risk and outcomes, as well as associations with potential genetic and lifestyle factors [4–6].

Archived patient tissue is an invaluable repository of information for epidemiological studies of DNA methylation. Much of this material is preserved using formalin fixation, and as a result, isolated DNA is usually fragmented and not suitable for array and sequencing based epigenomic analysis [4, 7, 8]. Thus, to date the use of genome-wide methylation analysis platforms has been limited by the availability of fresh frozen (FF) tissue. More recently approaches to restore fragmented DNA have offered promise for use of formalin fixed paraffin embedded (FFPE) tissue in large-scale epigenomic studies.

The goal of this study was to determine if restored FFPE DNA samples from breast cancer tissues allows reliable genome-wide DNA methylation analysis using the HumanMethylation450K Beadchip (450K). Because FFPE samples may be obtained on glass slides or in the form of punches or curls, we also determined the extent to which array-based methylation analysis depended on the type of FFPE tumor tissue. Lastly, we assessed the overlap of FF and FFPE DNA methylation levels in two large independent cohorts of breast tumors.

## RESULTS

### Intra-sample DNA methylation reproducibility between FF and FFPE tumors

We analyzed 10 patient tumors each supplied as four sample types: FF, FFPE slide, FFPE curl and FFPE punch. Patients tumor characteristics are summarized in Supplementary Table 1. The probe detection rate for all FF samples was greater than 99%. Of the restored FFPE samples, 9 of the 10 patient sample groups had greater than 98% CpG detection rate, with Tumor #2 slide (T2_S) and curl (T2_C) having the lowest detection rates at 80% (Supplementary Figure 2A). The low detection rate can be attributed to T2_S and T2_C high QCT values, 6.5 and 7.1, respectively. We determined efficient detection of FFPE samples was observed in all samples with a QCT ≤ 6 (Supplementary Figure 2B).

**Figure 1:**
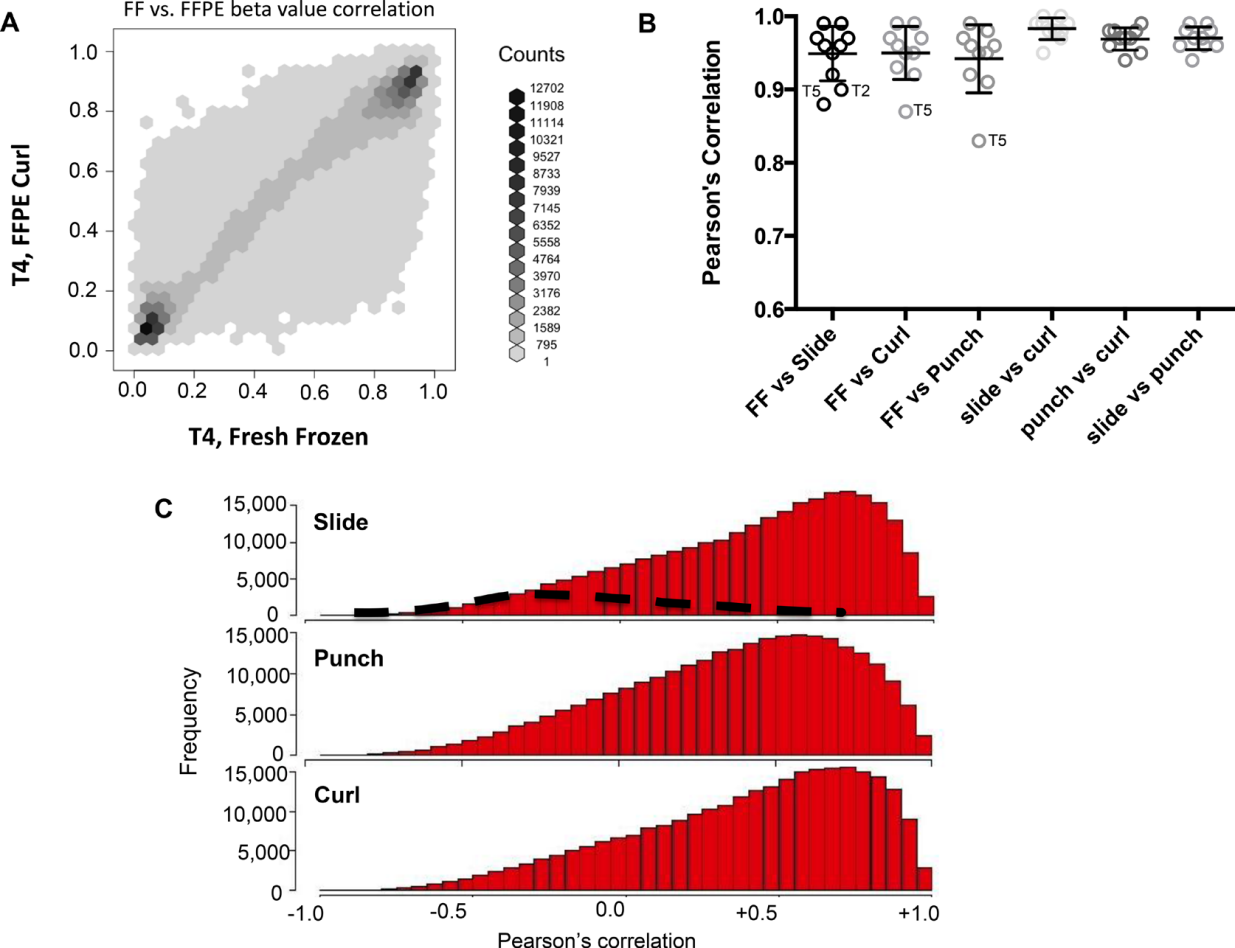
(**A**) The correlation between FF and FFPE probe values. (**B**) Correlation and standard error of mean β values between FF and each type of FFPE. (**C**) The top, middle and bottom histograms show the distribution of ρ values from FF-FFPE correlations for all loci in slide, curl and punch respectively. The black dashed line are locus specific correlations from Jasmine et al. analysis of 10 FF and FFPE colon tumor samples using the Illumina HumanMethylation27K and a homemade ligation method.

To determine the intra-sample consistency in DNA methylation levels between FF and FFPE derived DNA we used global, gene position (shore, shelf and island), locus specific correlations. Global specific correlations for each FF tumor sample was completed against each of the three FFPE tumor samples for a single patient using all filtered CpG sites. A dot matrix plot of FF β-values versus FFPE curl β-values for a representative sample (T4) is shown in Figure 1A (*ρ =* 0.96). Overall we observed good correlation between the FF and FFPE samples across all loci with a mean *ρ >* 0.95 (Figure 1B). Correlation was weakest for sample T5 despite good QCT values for the FFPE samples (Supplementary Figure 2B). The distribution of locus specific Pearson's correlation is shown in Figure 1C. There is a clear peak toward the right in all FFPE sample types suggesting overall high correlation at each locus compared in FF and FFPE samples. FF-FFPE correlation by shore, shelf and island are shown in Supplementary Figure 3. The correlation between FF and FFPE increases as the probe position shifts from shelf, *ρ =* 0.90, to island, *ρ =* 0.96, however the correlation across FFPE types shows little to no position dependent correlation change.

**Figure 2:**
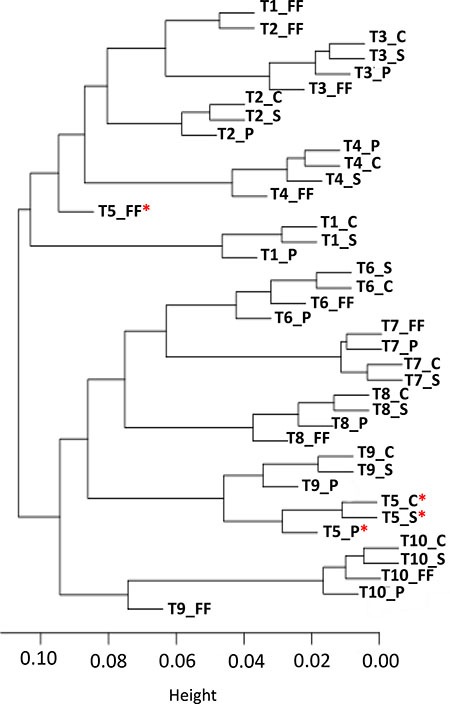
Hierarchical clustering analyses using all loci passing quality control shows clustering patterns of FF with FFPE counterparts, as well as the overall grouping pattern of all samples in two clusters, C1 and C2 *Imperfect clustering with FF counterpart (red) very poor clustering.

We further confirmed the intra-sample consistency within a single tumor by performing clustering analysis of matched patient tumors. Two distinct clusters were generated from unsupervised hierarchal clustering analysis using all filtered CpG sites (Figure 2). Except for T5, the FF and FFPE matched patient tumor sets of 9 patients were consistently grouped within the same cluster. For 6 of 10 patients (T3, T4, T6, T7, T8, and T10), the single FF and three FFPE samples clustered together such that they were the only sample group in a branching (Figure 2). The FF T5 sample was substantially separated from the three corresponding T5 FFPE samples in the dendogram. Analyses of the 65 SNPs provided in the 450K array [9] confirmed that all T5 samples were from the same patient (Supplementary Figure 4).

**Figure 3:**
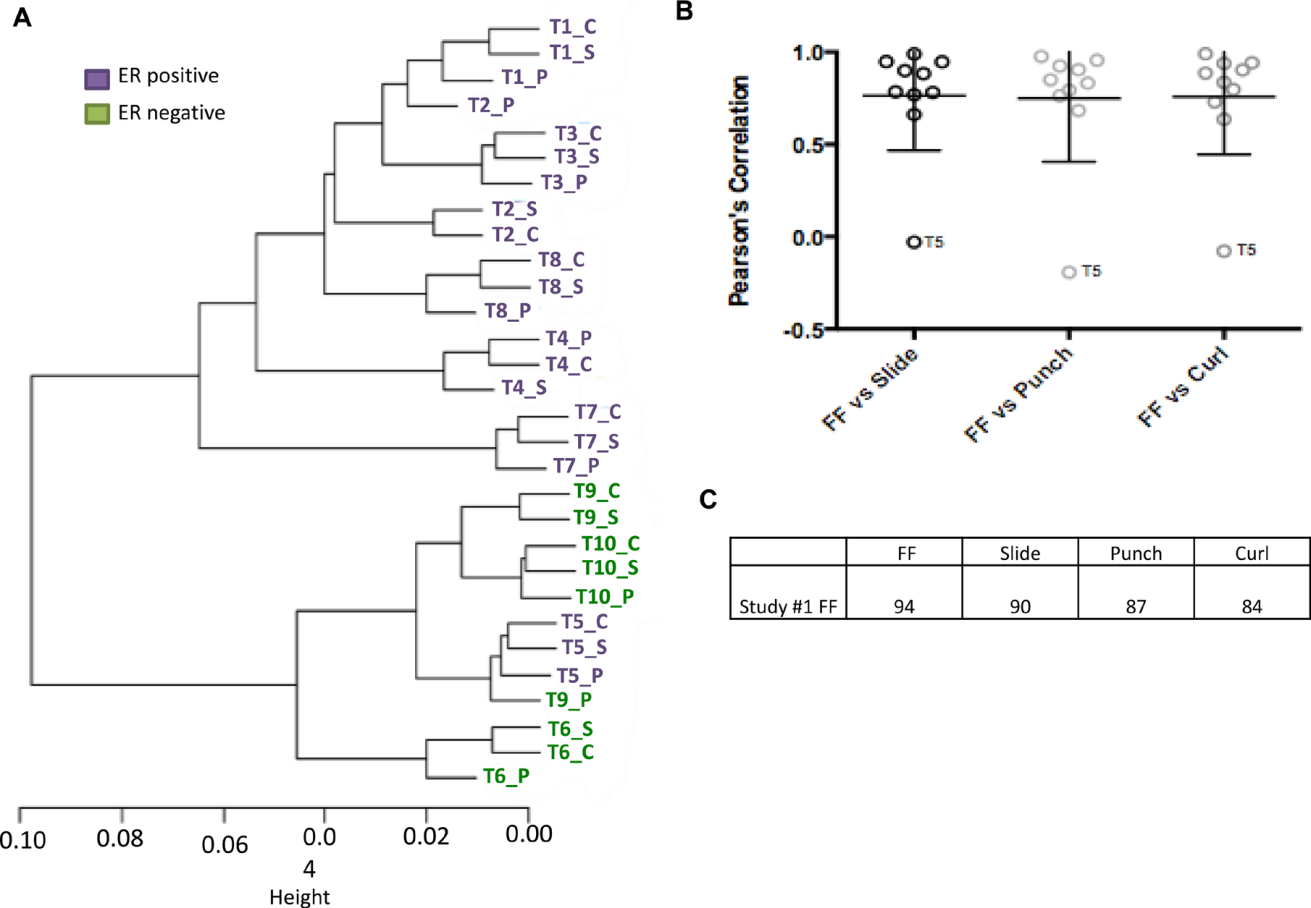
(**A**) Hierarchical clustering using 100 ER + and ER− DML identified in previous analyses of FF breast cancer tissues. (**B**) The FF and FFPE β-value correlations and standard error using these top 100 ER + and ER− DML are shown. (**C**) The percentage of overlap when comparing the top 100 DML identified in our previous study with all FF, FFPE curl, punch and slide DML found in the current pilot study.

Lastly, we determined the number of differentially methylated loci (DML) between ER+ and ER– tumors. Using Δβ > 0.17, we identified 21,925 DMLs between ER+ and ER– breast tumors in the FF breast tumors included in this study and 13,594 DMLs in the FFPE slide, 11,764 DMLs in the FFPE punch and 11,960 DMLs in FFPE curl breast tumors. Of the DMLs detected in FF, 73.2%, 58.3% and 65.5% were also identified as differentially methylated in FFPE slide, punch and curl, respectively. On the other hand, 45.4%, 31.3% and 35.7% of DML identified in FFPE slide, punch and curl respectively were identified as differentially methylated in FF samples. The 100 loci that are most differentially methylated between ER+ and ER– tumors in our previous FF study [17] successfully segregated the slide, curl and punch FFPE samples by ER status; however, as with clustering using all loci, sample T5 remained an outlier even when using these 100 (Figure 3A). The correlation of β-values of FF tissue with FFPE counterparts using loci found to differentiate sample by ER status in FF tissue is shown in Figure 3B. The mean FF-FFPE correlation was strong, ranging between .75 and .76 for all three FF-FFPE pairings, even when include T5. In all cases, ≥ 84 of the top 100 loci from the larger FF study were also significantly differentially methylated between ER+ and ER– tumors in each of the four sample types (Figure 3C).

### FFPE DNA methylation reproducibility in FF across two large independent cohorts

We previously reported 134 loci differentially methylated using a Δβ > 0.17 in the FF cohort [10]. In the larger WCHS FFPE cohort we identified 45 DMLs (Δβ > 0.17) (AC Espinal et al. submitted, *Journal of the National Cancer Institute*). A large majority of DMLs (39/45, 87%) found in the FFPE samples were among the 134 DMLs identified previously in the FF study, 38 of these 39 were in the top 100 FF DML when ranked by the greatest Δβ-value. Assuming FF DML reflect true methylated regions, the proportion of DML detected in the FFPE cohort and in FF DMLs indicate a very good positive predictive value, PPV = .87 (95% CI .73, .95). When shifting the Δβ down to > 0.10, the percentage of DMLs overlapping between studies was reduced (49%), showing the expected tradeoff in PPV and sensitivity (Table 1).

**Table 1:** Proportion of FFPE DML identified as DML in FF (positive predictive value) and sensitivity

Measurements comparing FFPE and FF DML	Δβ-value threshold cutoffs for FFPE DML , FF DML			
	0.17, 0.17	0.10, 0.10	0.10, 0.17	0.17, 0.10
**Sensitivity (95% CI)**	0.29(0.22, 0.38)	0.08(0.07, 0.09)	0.72(0.63, 0.79)	0.02(0.01, 0.02)
Positive Predictive Value* (95% CI)	0.87(0.73, 0.95)	0.49(0.44, 0.53)	0.23(0.19, 0.28)	0.96(0.85, 0.99)

*Proportion of FFPE DML identified in WCHS cohort as DML in FF. Using a Δβ-value threshold of .17 in FFPE a total of 45 DML were identified and using Δβ-value = .10 a total of 410 DML were identified.

Table 2 shows the number of FFPE DMLs identified in our previous FF analyses with respect to ER status (ER–, ER+, and ER independent). We again see that loci in FFPE are consistently detected in FF, however as before the sensitivity is poor (data not shown). ER negative subtype had considerably lower overlap across all Δβ-value thresholds (Table 2).

**Table 2:** Proportion of FFPE DML identified as DML in FF methylation analyses

Proportion of FFPE DML identified as DML in FF (PPV) by Tumor Type	Δβ-value threshold cutoffs for FFPE DML, FF DML			
	0.17, 0.17	0.10, 0.10	0.10, 0.17	0.17, 0.10
**ER+**	8/10(80)	30/36(83)	11/36(31)	10/10 (100)
**ER independent**	25/2696)	105/114 (92)	74/114 (65)	26/26 (100)
**ER–**	6/9(67)	64/260(25)	11/260(4)	6/9(67)

### WCGNA module preservation across FF and FFPE

**Figure 4:**
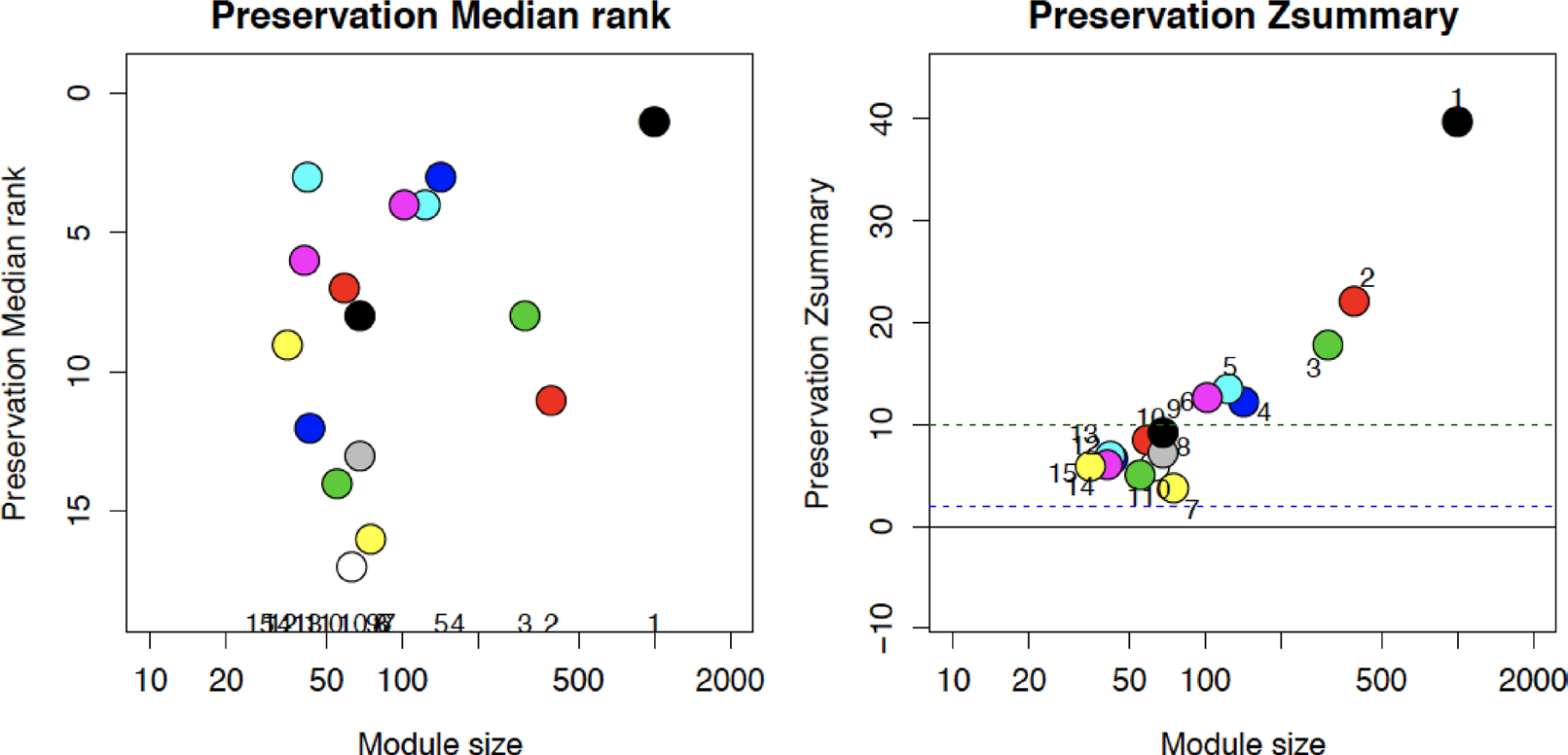
Unique DML (*n* = 2664) from FF and FFPE analyses were identified and used to estimate the number of modules in FF tissue; the same loci modules were then created in the full FFPE data set Preservation between the modules is shown by Preservation Median Rank (left) and the Zsummary (right) with module size (number of genes) shown on the x-axis in both left and right figures. The preservation rank, how well a module was preserved from FF to FFPE compared to other modules, and the Zsummary, how well each module is preserved, with values ≥ 10 indicating evidence of preservation, are the y-axis on the left and right figure respectively.

The results of module preservation analysis are shown in Figure 4. The left and right panels show the results for median rank and Z-summary statistics of module preservation of FF modules in all FFPE data, respectively. From Figure 4 we see that the largest modules (those with the most number of genes) are preserved across FF-FFPE. Specifically, of the 2664 loci included almost 85% (*n* = 2246) of the loci (contained in modules 0, 1, 2, 3, 5, 6) were preserved across FF-FFPE irrespective of FFPE type (slide, curl or punch).

## DISCUSSION

In this study, we measured and correlated DNA methylation profiles in restored DNA from FFPE (slide, punch and curl) and paired FF breast cancer tissue, as well as compare DML between two separate large FF and FFPE cohorts. All restored FFPE DNA with a QCT value ≤ 6 provided acceptable overall detection of CpG sites. The intra-sample analyses of 10 matched breast tumor samples showed that global and locus specific correlations of beta values were strong between FF and restored FFPE samples and the WCGNA analyses further confirm there is good agreement between FF and FFPE. Clustering analysis suggested that FF and restored FFPE tumors from a single patient tend to group together. In contrast Jasmine et al., who analyzed methylation of 10 colon cancer samples using Illumina Human Methylation 27K array and found poor locus-specific β-values correlations when comparing FF colon tumor samples with FFPE samples restored using a ligation based restoration protocol [7, 8]. Our results highlight the importance of repairing fragmented FFPE DNA by Illumina's chemical restoration protocol prior to whole-genome amplification. In addition to our analysis, two recent studies carried out similar analyses and also determined efficient CpG detection of FFPE tumors on 450K array after Illumina's restoration protocol in multiple cancers [9, 11].

Although good overall intra-sample correlations were seen, sample T5, the only grade IIIB tumor, showed poor FF/FFPE clustering and beta value correlation. This finding perhaps reflects the impact of tumor heterogeneity on methylation patterns in FF and FFPE tissues; for example, it is also possible that the poor correlation and lack of clustering are due to differences in the proportion of tumor tissue in the FF and FFPE samples [12]. The age of the tumor block, has been shown to negatively affect the reproducibility of DMLs detected in DNA from matched FF and FFPE samples [12]. In the present study most of FFPE samples were prepared within a 2-year window and thus the impact of block age should be significantly less. The comparison between FFPE slide, curl and punch methylation patterns versus FF patterns consistently showed better correlation between slide and curl methylation patterns and those of FF versus FFPE punch; this finding may be explained by the latter being more localized sample and thus this difference may speak to tumor heterogeneity. These findings indicate that slide and curl sections are more representative of the entire tumor cell population whereas the punch is more specific to the epithelial compartment which is ultimately reflected in the replication of DMLs found in FFPE studies.

Our results were similar to Dumenil et al., who compared methylation patterns in DNA from FF colon cancer tissues with Illumina restored DNA isolated from matched FFPE samples using the 450K platform, with respect to clustering and number of loci replicated [12]. However, they utilized a *p-value* cutoff to determine DMLs shared between tissue types. We conducted a more in depth comparison of a large FF DML [10] and FFPE DML validation cohorts using different beta value thresholds that were informative for study design considerations. Overall DML identified in FFPE were also identified in FF, however based on our findings it appears analyses of FFPE tissue would not be as sensitive as FF. This essentially translates to lower power to detect loci but a reasonably good Type II error. Lowering the FFPE Δβ ≥ 0.10 identified more DML, many of which may not be true DMLs (assuming the gold standard is FF), however it will also capture many more of the loci that are detected in analyses of FF tissue. This area of Δβ-value thresholds for FFPE merits further exploration. Interestingly, regardless of β-value threshold, the loci differentiating ER–negative subtype were less likely to replicate. The marked decrease in overlap between ER–negative tumors may partially reflects the heterogeneity of ER–negative tumor subtype, suggesting that methylation patterns show more variation in ER–negative breast cancer, further highlighting the need to better understand this diverse tumor type. This variation in the ER phenotype means power must be carefully considered for ER–negative methylation experiments.

This is only the third pilot investigation comparing methylation in FF-FFPE samples [8, 9, 12] and the second to imply using both FF and FFPE separately is a viable option to investigate epigenetics in large populations. We report that restored FFPE DNA will run efficiently on the 450K array but more importantly will replicate in independent cohorts of FF samples as well suggest that the current Δβ cutoff of 0.17, based on FF tissue, should be carefully considered for analyses of FFPE methylation data. We suggest using a stringent (Δβ ≥ 0.17) β-value threshold in FFPE studies when detection of fewer but highly replicated DMLs is favorable. However, to generate a more comprehensive list of loci a relaxed (Δβ ≥ 0.10) FFPE β-value threshold is more desirable. Given the small studies to date and the large amount of archival tissues available additional larger scale studies of the behavior of methylation in FF and FFPE across diseases and within disease subtypes are imperative.

## MATERIALS AND METHODS

### Intra-sample patient cohort

We compared methylation patterns of FF and FFPE derived DNA from 10 matched patient samples. All women had undergone surgery for breast cancer at Roswell Park Cancer Institute (RPCI) and gave informed consent allowing use of any tissue remnants banked for research. Breast tumor tissue was reviewed by a pathologist at RPCI's Pathology Resource Network (PRN), snap frozen in liquid nitrogen, and stored at –80^°^C. Remaining surgical tissues were fixed in 10% formalin and paraffin embedded. FF samples were matched with FFPE samples from the same patients, and were provided in the form of one 10 mm punch, one 20 μm curl and four 10 μm sections on slides for a total of 40 samples.

### DNA preparation

Genomic DNA from FF breast tumors was isolated using the Puregene (Gentra D70KA) DNA purification protocol, as per manufacturer's instructions. Matched FFPE samples were deparaffinized in xylene, lysed in a TNES Buffer (10 mM Tris, 150 mM NaCl, 2 mM EDTA and 0.5% SDS) with 20 mg/mL Proteinase K, and incubated at 56°C with constant rotation until completely digested. Lysates were then heated at 70°C for 20min to inactivate the Proteinase K and stored at 4°C. DNA from a 5ul aliquot of FFPE lysate was purified using the DNA Clean & Concentrator-5 kit (Zymo Research) for quantification by Quant-iT Picogreen dsDNA assay kit (Invitrogen).

### Quality control (QC) assay of FFPE samples

Real-time PCR assays were run in triplicate using DNA isolated from FFPE samples and a QC standard, using primers supplied in the Illumina Infinium HD FFPE QC Kit (Infinium HD FFPE QC Assay Protocol, Illumina). The quality cycle threshold (QCT) value was calculated by subtracting the average Cq of Illumina QC standard from the average Cq value determined for each FFPE sample. Illumina recommends that a QCT value ≤ 5 be utilized for optimal assay performance.

### Genome-wide methylation analysis using the Illumina 450K Beadchip

Prior to hybridization to the 450K Bead Chip, 1μg DNA from FF samples or 1μg DNA equivalent of FFPE lysate, as quantified by Quant-iT Picogreen dsDNA assay kit (Invitrogen), was treated with sodium bisulfite using the Zymo EZ DNA methylation kit, as instructed. Following bisulfite treatment, FFPE lysate samples were restored using the Infinium HD DNA Restoration Kit (Illumina), which implements two enzymatic reactions and an optimized whole genome amplification step. Samples were randomized with respect to sample type, patient age and estrogen receptor (ER) status (13) across four 450K BeadChips. Hybridized and processed arrays were scanned using Illumina BeadArray Reader with High-Density (HD) Technology and BeadScan software. The raw intensity was then extracted using the GenomeStudio module.

### Statistical analysis and quality control

The raw data from GenomeStudio was summarized into BeadStudio IDAT files, and processed by the R package *minfi*. The 450K array data were subjected to rigorous sample and locus specific quality control criteria (Supplementary Figure 1). The data were normalized by SWAN normalization, and batch effects corrected using the ComBat algorithm (14–16). Low quality probes (detection *p-value* > 0.05 in more than half of samples) and samples with detection *p*-values < 1 × 10^–5^ at more than 75% of CpG loci were removed from the analysis using the *IMA* package [13]. Probes containing SNPs and/or probes that map ambiguously were excluded [17–19]. After the pre-processing, the final dataset contain 276,149 probes across 40 samples.

### Intra-sample correlations

For each locus, the methylation level was denoted by a beta value (β) ranging from 0 (unmethylated) to 1 (100% methylated). To measure the sample correlation between FF DNA samples and FFPE samples, Pearson's correlations (*ρ*) were calculated using β-values from two sets of loci: (1) all loci passing quality control and (2) the 100 loci which were identified to be most differentially methylated between ER + and ER– tumors in our previous study on a large population of FF breast tissues [10].

### Clustering and differential methylation analysis

To determine the similarities of FF and FFPE samples, unsupervised hierarchical clustering analysis based on the average linkage of Pearson correlation was performed using all loci. In addition, the clustering analysis was applied on the previous 100 loci to examine if their ability of distinguishing tumor ER status in FF samples[17] retains in FFPE samples. Differentially methylated CpG loci (DML) between interested groups were defined as those with a mean β-value difference (|delta β|) of greater than 0.17, as recommended by Illumina [15,16]. Statistical significance was evaluated using a Bayesian based *t-test* by the linear model for each comparison.

### Validation cohorts

We utilized two large independent cohorts to investigate the relationship of methylation measurements in FF and FFPE tissue: a cohort of 138 FF breast tumors previously described (10) and a cohort of 733 FFPE patient samples obtained through the Women's Circle of Health (WCHS), a case-control study of aggressive breast cancer in African-American and European-American women [20–22]. The FFPE cohort DNA was extracted from either a 10 mm punch, 20 μm curl or 10 μm slides and all samples underwent QC assay as described above. The Illumina 450K Beadchip was run on both the FF and FFPE samples, with raw data processed as described above. With filtered datasets, we then compared the total number of race-associated DMLs (AA vs EA) between the FF and FFPE cohorts, and further examined DMLs by ER–status (ER+, ER–) using two different delta-β-value thresholds (Δβ > 0.10 and Δβ > 0.17) yielding a total of 12 comparisons. The EpiR package was used to determine the sensitivity, specificity, and positive and negative predictive values assuming FF cohort DMLs as the gold standard.

### Weighted gene correlation network analyses (WCGNA)

The function modulePreservation in WGCNA R package was used to assess the preservation of FF modules in the FFPE data [23]. A total of 2664 unique DML from FF and FFPE analyses were identified and used to estimate the number of modules in FF tissue. Using the same loci modules were then created in the full FFPE data set, as well as slide, curl and punch datasets separately. Median rank and Z-summary statistics of module preservation of FF modules in each FFPE dataset were determined. The permutation based Z-summary statistic reflects module preservation between FF and FFPE, such that the higher the *Z*-value the stronger the evidence that the module is preserved across datasets. *Z* values >10 indicate moderate preservation, while values below 10 indicate no evidence of preservation.

## SUPPLEMENTARY MATERIALS FIGURES AND TABLES


