# Supplementary material for: A methodological study of genome-wide DNA methylation analyses using matched archival formalin-fixed paraffin embedded and fresh frozen breast tumors
Source: Oncotarget. 2017 Jan 19;8(9):14821–9. doi: 10.18632/oncotarget.14739 (PMC5362446; doi:10.18632/oncotarget.14739)
Supplement: Supplementary file 1 [file oncotarget-08-14821-s001.pdf]

A methodological study of genome-wide DNA methylation analyses using matched archival formalin-fixed paraffin embedded and fresh frozen breast tumors

Supplementary Materials

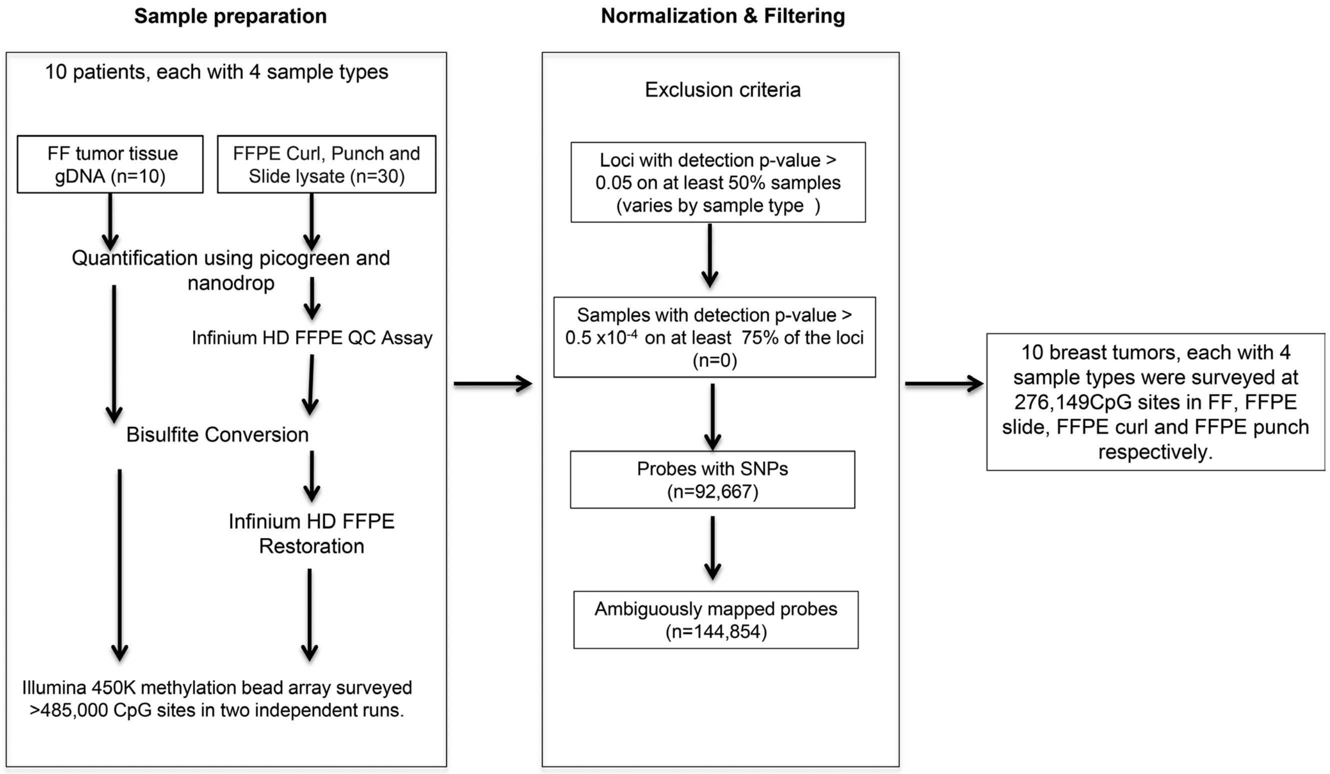

Supplementary Figure 1: Sample and data generation schema.

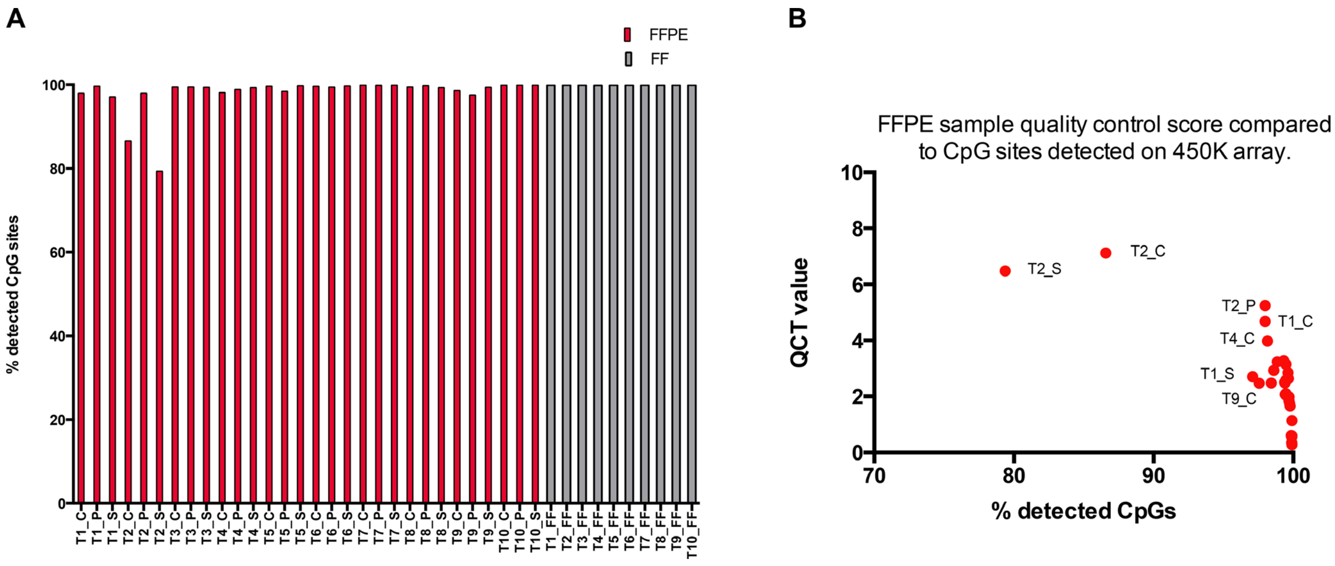

Supplementary Figure 2: (**A**) Percentage of CpGs detected ($p < .05$) for all sample types. (**B**) The FFPE sample QCT value (see methods) is compared to the percentage of CpG sites detected on the array.

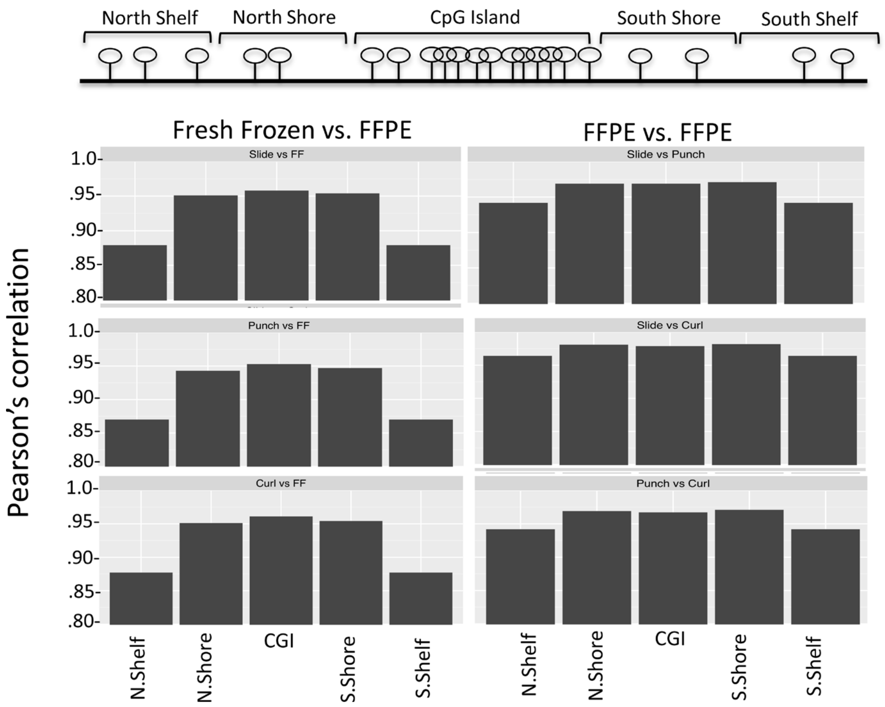

Supplementary Figure 3: The ideogram across the top of the histograms represents the position of the shelves, shores and islands. The 5 histograms in each of 6 graphs below the ideogram correspond to the labeled North Shelf, North Shore, Island, South Shore and South Shelf. The left column of histograms shows methylation probe correlations by position for FF vs. FFPE slide, curl and punch (top left to bottom left, respectively). The right column shows pairwise CpG methylation probe correlations across the three FFPE types.

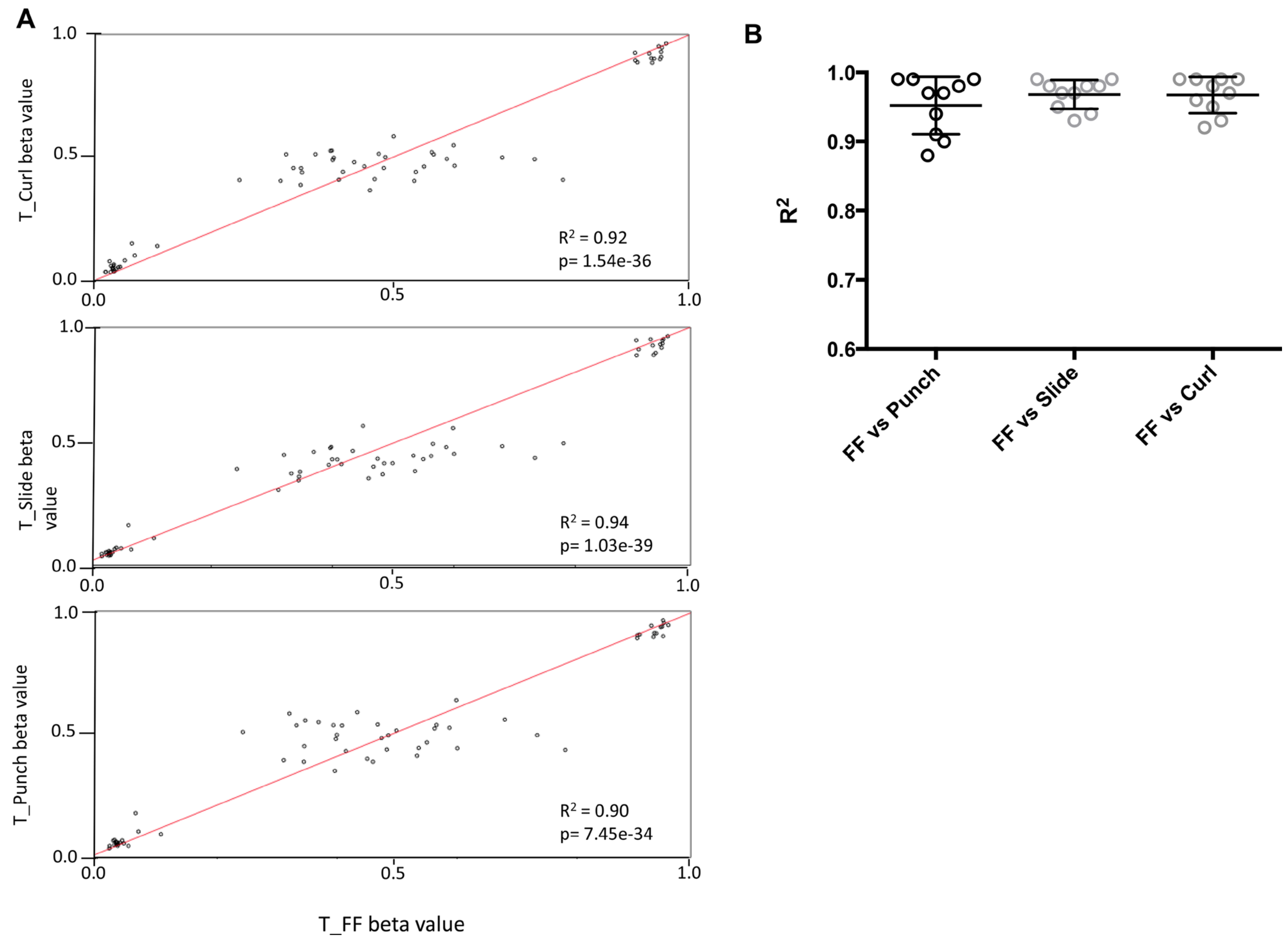

Supplementary Figure 4: Sample genotyping using the 65 illumina SNP probes included on the 450K confirmed all matched patient samples were correct. (**A**) An example for the poorly cluster T5_FF compared to the 3 matched FFPE samples (T5_Curl, T5_Slide, T5_Punch). The beta values for the 65 SNP probes are plotted and correlation and p-values are shown for each comparison. (**B**) A summary of correlations for all matched samples.

Supplementary Table 1: Breast cancer patient characteristics

Tumor ID	Age at Diagnosis	Year of Fixation	Race	ER, PR, & HER2 status	Tumor Grade
T1	60	1998	European American	+/+/−	III
T2	52	2000	European American	+/+/−	II
T3	68	1996	European American	+/+	I
T4	33	1996	European American	+/+	III
T5	70	1998	European American	+/−/−	III
T6	79	1997	European American	−/−	III
T7	50	2010	African American	+/+/−	III
T8	46	1996	African American	+/−/−	II
T9	83	1999	European American	−/−/−	III
T10	43	2011	African American	−/−/−	III
